# The Insulin Treatment Satisfaction Questionnaire and Assessment of Satisfaction with a Latest-generation Insulin Pump

**DOI:** 10.17925/EE.2015.11.02.67

**Published:** 2015-08-19

**Authors:** Michael Bromba, Fiona Campbell, Brian L Levy

**Affiliations:** 1. Froidevaux & Partner GmbH, Zurich, Switzerland; 2. Leeds Teaching Hospitals, Leeds, UK; 3. LifeScan Inc., Wayne, Philadelphia, US

**Keywords:** Type 1 diabetes, type 2 diabetes, Insulin Treatment Satisfaction Questionnaire, latest-generation insulin pump, CHOICE study, treatment satisfaction

## Abstract

Satisfaction with the latest-generation insulin pump (LGIP) was assessed in patients with diabetes mellitus enrolled in the Comparing Perception of Insulin Therapies for T1D Patients with the Aim to Improve Quality of Care (CHOICE) study. The Insulin Treatment Satisfaction Questionnaire (ITSQ), a measure of insulin treatment satisfaction, together with additional questions assessed respondents’ perceptions of glucose control, their satisfaction with major LGIP features and preference for the LGIP versus their previous treatment, was used. The LGIP (Animas^®^ Vibe™) was considered to be a better method for delivering insulin compared with their therapy before switching and was rated high for treatment satisfaction. These findings should be useful to clinicians when considering the possibility of transferring a patient from their existing treatment regimen to a LGIP.

The global incidence of diabetes mellitus (DM) is rapidly increasing. In 2014, 387 million people were living with diabetes representing one in 12 of the population and this number is estimated to increase by 205 million by 2035. There were 4.9 million deaths of people living with diabetes in 2014 equivalent to one person dying every 7 seconds.^1^

Insulin is the most common treatment in type 1 diabetes (T1D) patients, while most patients with type 2 diabetes (T2D) receive oral treatments although many are treated with insulin. There are a large variety of treatment methods available for delivering insulin, including syringe and vial, pre-filled insulin pens, inhaled insulin and insulin pumps. Both intranasal and transdermal routes have been investigated, but proved to be ineffective. In view of the many treatment options available, it is important that patient satisfaction with their treatment can be accurately and reliably assessed. A study to investigate the association between quality of life and treatment adherence in T1D indicated that patients with a greater perceived ease of adherence to a treatment regimen reported a higher level of quality of life.^2^ Patients with high satisfaction with their treatment may be more likely to be more compliant with it and have improved clinical outcomes.

The Insulin Treatment Satisfaction Questionnaire (ITSQ), a measure of insulin treatment satisfaction, was developed by Anderson et al. to be applicable to a wide range of insulin therapies.^3^ It was initially validated in 572 patients with T1D and T2D to produce a five-factor, 22-item instrument, which has proved to be a conceptually and psychometrically sound and valid measure.

In this article, we discuss the ITSQ and its use to assess satisfaction with the Animas^®^ Vibe™ insulin pump, a latest-generation insulin pump (LGIP), which is continuous glucose monitoring (CGM)-enabled, in patients recruited for the Comparing Perception of Insulin Therapies for T1D Patients with the Aim to Improve Quality of Care (CHOICE) study as recently published by Barnard et al.^4^

## Development of the Insulin Treatment Satisfaction Questionnaire

A total of 572 T1D or T2D patients were involved in the development of the ITSQ, recruited from three US medical centres.^3^ Of these, >80 % had T2D and 78 % were administering insulin with syringe and vial, 13.5 % by pen injection, 4.7 % by pump and 0.9 % by inhaler. The ITSQ was validated in these patients and confirmatory factor analysis produced a five-factor structure based on a total of 22 items asked: satisfaction with insulin delivery device, glycaemic control, hypoglycaemic control, convenience of regimen and lifestyle flexibility. The confirmatory psychometric validation in 402 patients showed that test-retest reliability over approximately 2 weeks was very good for the 22 items in total (0.90) and confirmatory psychometric validation indicated that internal consistency reliability measured by Cronbach α coefficient was excellent (0.93). The patients studied confirmed the composition of the final ITSQ as 22 items in five content clusters. All subscales of the final ITSQ were transformed from their original scale (0 to 132) to a scale of 0 to 100, in which 100 indicates complete satisfaction to insulin treatment (i.e. the higher the score the better the treatment satisfaction).

**Table 1:** Mean ITSQ Subscale Score by Patient Age for All Countries

Mean ITSQ Score of Subscale*					
Age (years)	Insulin Delivery Device Satisfaction (Range 0–36)	Hypoglycaemic Control (Range 0–30)	Glycaemic Control (Range 0–18)	Inconvenience of Regimen (Range 0–30)	Lifestyle Flexibility (Range 0–18)
12–17	26.0	19.8	11.0	18.1	12.9
18–29	25.9	19.0	10.6	18.3	12.1
30–49	30.4	21.0	12.0	20.4	11.9
50–64	31.4	22.9	12.6	22.3	12.4
65+	34.2	25.2	15.3	26.4	13.4

* The higher the better (e.g. if the subscale score range is 0–18, 18 is the highest score that can be reached). ITSQ = Insulin Treatment Satisfaction Questionnaire. Source: Froidevaux & Partner, 2014.

**Table 2:** Treatment Satisfaction Scores in the Five ITSQ Subscales

Subscale	Mean Treatment Satisfaction Score (Scale Range)
Insulin delivery device satisfaction	29.5 (scale 0–36)
Glycaemic control	12.0 (scale 0–18)
Hypoglycaemic control	21.1 (scale 0–30)
Convenience of regimen	20.4 (scale 0–30)
Lifestyle flexibility	12.3 (scale 0–18)

Insulin Treatment Satisfaction Questionnaire (ITSQ) scores among all respondents in the five subscales are in the format the higher-the-better using the original scales. Reproduced with permission from: Barnard, 2015.^4^

Interestingly, patients with higher compliance (no missed insulin doses during the last 4 weeks) had lower glycated haemoglobin (HbA_1c_) values, fewer symptoms and higher ITSQ satisfaction scores compared with those with partial compliance, who missed some doses (p=0.05; p=0.001; and p=0.003, respectively). ITSQ proved to be a well-accepted instrument since there were less than 5 % missing values in the samples and clinically meaningful as it could discriminate between various factors such as method of insulin administration and patient adherence. Furthermore <5 % missing values were seen, indicating the ITSQ is highly practical measure.

## Treatment Satisfaction with the Latest-generation Insulin Pump System Assessed by Insulin Treatment Satisfaction Questionnaire

It is critical for healthcare professionals and medical device suppliers to accurately gauge DM patient attitudes and responses to insulin pumping systems in order to fully determine their efficacy and utility. Knowing which device provides optimal therapy is valuable information and could help stem the increasing burden of this disease. In order to address this, the CHOICE study was designed as a multicentre, non-interventional, cross-sectional study of T1D patients who had recently transitioned from either multiple daily insulin injections (MDIs) or older-generation insulin pumps to a LGIP (CGM-enabled) system (Animas^®^ Vibe™ LifeScan, Wayne, US).^4^ At each study site, clinicians were provided with pre-formatted e-mails to invite the participants. Prospective respondents to the e-mail, were redirected to the study server in order to complete the online survey having given written, informed consent. The online survey was a 50-item questionnaire that included the original ITSQ scale^3^ in its country-specific validated form to measure overall insulin treatment satisfaction as well as its underlying dimensions. Additional questions assessed respondents’ perceptions of glucose control, their satisfaction with major LGIP features and preference for the LGIP versus their previous treatment. Glycaemic status was assessed by self-reported fasting blood glucose data.

## Patient Demographics in the CHOICE Study

In total, 356 T1D patients recruited from four language areas in five EU countries (France, Germany, The Netherlands and UK/Ireland) completed the questionnaire. Patients were recruited to two study arms: insulin pump experienced and pump naïve. Pump-experienced patients had been treated by insulin pump systems of Animas^®^, Medtronic^®^, Roche^®^ or other and had to be on the LGIP for at least 3 months. Patients who had not previously used an insulin pump had either self-treated with insulin pens, vials and syringes or had been untreated. MDIs were being used by 61 % of patients prior to changing to the LGIP. The mean age of the patients was 38.4 years (SD 16.1) and ranged from 12 to >65 years (*Table 1* shows the age breakdown).

## Treatment Satisfaction in the CHOICE Study

Treatment satisfaction with the LGIP was high with the mean overall ITSQ score 95.1 among respondents regardless of their previous insulin delivery system. There were no significant mean differences between patients previously using a pump or MDIs injections. In addition, in each of the five subscales, treatment satisfaction scores were high (see *Table 2*).

**Figure 1:**
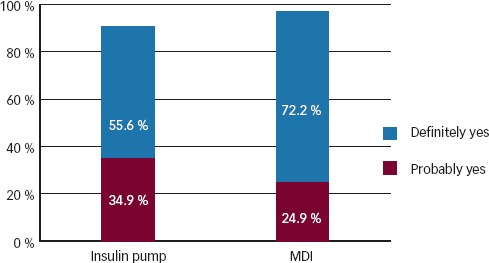
Recommendation of the LGIP by Former Treatment (%) LGIP = latest-generation insulin pump; MDI = multiple daily insulin injections.

Most patients reported that they would definitely (66 %) or probably (28 %) recommend the LGIP to other people with T1D. Patients on former MDI treatment would definitely (72 %) or probably (25 %) recommend the LGIP (see *Figure 1* – possible answers were: definitely not; probably not; probably yes; definitely yes).

## Patient Satisfaction with Latest-generation Insulin Pump Features

Overall, more than 90 % of respondents felt that the ease of use of the LGIP, giving a bolus at meal time, the warnings and alarm safety system were important and most patients were satisfied with these features.

When the ITSQ subscales were evaluated by age group, it was found that the older the patients the higher the satisfaction with the LGIP as a delivery method (p<0.001), its ability to control hypoglycaemia and/ or glycaemia (p=0.001) and its convenience (p<0.001). See *Table 1*, for results of a series of Analysis of Variance (ANOVA).

In-depth analysis evaluated whether a patient’s satisfaction with the essential features of a LGIP affected core dimensions of the overall treatment satisfaction. *Table 3* summarises whether patient reported satisfaction with specific LGIP features increases the ITSQ subscale scores. Mean subscale differences between patients who were satisfied and patients who were less satisfied with a given feature were tested by independent samples t-tests. The ease of customising profiles such as for basal rates or for exercise was rated highly on insulin delivery device satisfaction, glycaemic control and convenience of the regimen (p<0.01) and hypoglycaemic control (p<0.05). Satisfaction with the warnings and alarm safety system accounted for highly significant higher mean scores on most of the ITSQ subscales (all p<0.01).

**Table 3:** Comparison of LGIP features with ITSQ subscales

Feature	Insulin Delivery Device Satisfaction	Hypoglycaemic Control	Glycaemic Control	Inconvenience of Regimen	Lifestyle Flexibility
Ease of use (e.g. menu, navigation, instructions, etc.)	Yes (*)				
High-contrast colour screen				Yes (*)	Yes (**)
Weight	Yes (***)			Yes (**)	
Size	Yes (***)	Yes (***)		Yes (***)	
Warnings and alarm safety system	Yes (***)	Yes (***)	Yes (***)	Yes (***)	
Ease of customising profiles (e.g. for basal rates, exercises, sick days, etc.)	Yes (***)	Yes (**)	Yes (***)	Yes (***)	
Low basal increment of 0.025 U	Yes (**)		Yes (*)		
Low bolus increment of 0.05 U	Yes (**)		Yes (**)		
Giving a bolus at meal or snack time	Yes (***)	Yes (**)	Yes (**)		
ezBG feature that automatically calculates a correction bolus dose	Yes (*)	Yes (**)	Yes (**)	Yes (**)	
Continuous glucose monitoring option				Yes (*)	

Independent sample T-tests: *** p<0.01; ** p<0.05; * p<0.1. ITSQ = Insulin Treatment Satisfaction Questionnaire; LGIP = latest-generation Insulin pump.

Significant mean differences in most of the ITSQ subscales were also evidenced for the ezBG feature that automatically calculates a correction bolus dose. The ability to give a bolus at meal or snack times also showed high satisfaction values for insulin delivery device satisfaction, glycaemic control and hypoglycaemic control (p<0.01; p<0.05; and p<0.05, respectively).

## Conclusion

Adherence and thus improved glycaemic control are influenced by an insulin treatment regimen that complements patient’s expectations and lifestyle demands. In order to compare different insulin modes of delivery, an instrument that is clinically meaningful and psychometrically sound is needed. The ITSQ satisfies these requirements and the low incidence of missing values in the preliminary and confirmatory validation illustrates its utility in T1D patients. The instrument appears to be clinically meaningful and reproducible with convergent validity with overlapping instruments.

In the CHOICE survey of patients with T1D, using the ITSQ and additional questions to determine treatment satisfaction in those using an Animas^®^ Vibe™ LGIP system, patients recorded higher satisfaction scores regardless of previous insulin therapy or age. This finding might predict better glycaemic control. Furthermore, satisfaction in terms of delivery method, hypoglycaemic and glycaemic control and convenience of the regimen with the Animas^®^ Vibe™ LGIP was higher in older patients.

This is an interesting finding and may indicate that older patients welcome newer technologies to manage their DM. Features such as the automatic calculation of correction boluses, a warnings and alarm safety system and the ease of customising profiles (e.g. basal rates for sick days, for exercises) proved to affect directly the core dimensions of T1D treatment satisfaction (glycaemic and hypoglycaemic control or convenience of regimen), which in turn may positively influence a patient’s treatment adherence.

These results from the CHOICE study indicate that T1D patients considered that the Animas^®^ Vibe™ LGIP was a better method for delivering insulin compared with their therapy before switching and rated it high for treatment satisfaction. These findings should be useful to clinicians during discussions with a patient on the possibility of changing to a LGIP from their existing treatment regimen.
